# Visualization of molecular fluorescence point spread functions via remote excitation switching fluorescence microscopy

**DOI:** 10.1038/ncomms7287

**Published:** 2015-02-17

**Authors:** Liang Su, Gang Lu, Bart Kenens, Susana Rocha, Eduard Fron, Haifeng Yuan, Chang Chen, Pol Van Dorpe, Maarten B. J. Roeffaers, Hideaki Mizuno, Johan Hofkens, James A. Hutchison, Hiroshi Uji-i

**Affiliations:** 1 KU Leuven, Department of Chemistry, Celestijnenlaan 200G-F, B-3001 Heverlee, Belgium; 2 IMEC, Kapeldreef 75, B-3001 Heverlee, Belgium; 3 KU Leuven, Department of Physics and Astronomy, Celestijnenlaan 200D, B-3001 Heverlee, Belgium; 4 KU Leuven, Department of Microbial and Molecular Systems, Center for Surface Chemistry and Catalysis, Kasteelpark Arenberg 23, B-3001 Heverlee, Belgium; 5 University of Copenhagen, Nano-Science Center/Department of Chemistry, Universitetsparken 5, 2100 Copenhagen, Denmark; 6 ISIS & icFRC, Université de Strasbourg & CNRS UMR 7006, Strasbourg 67000, France; 7 School of Chemistry and Bio21 Institute, University of Melbourne, Victoria 3010, Australia; 8 PRESTO, Japan Science and Technology Agency (JST), 4-1-8 Honcho Kawaguchi, Saitama 332-0012, Japan

## Abstract

The enhancement of molecular absorption, emission and scattering processes by coupling to surface plasmon polaritons on metallic nanoparticles is a key issue in plasmonics for applications in (bio)chemical sensing, light harvesting and photocatalysis. Nevertheless, the point spread functions for single-molecule emission near metallic nanoparticles remain difficult to characterize due to fluorophore photodegradation, background emission and scattering from the plasmonic structure. Here we overcome this problem by exciting fluorophores remotely using plasmons propagating along metallic nanowires. The experiments reveal a complex array of single-molecule fluorescence point spread functions that depend not only on nanowire dimensions but also on the position and orientation of the molecular transition dipole. This work has consequences for both single-molecule regime-sensing and super-resolution imaging involving metallic nanoparticles and opens the possibilities for fast size sorting of metallic nanoparticles, and for predicting molecular orientation and binding position on metallic nanoparticles via far-field optical imaging.

## Abstract (web summary)

Plasmonic nanoparticles can dramatically enhance the optical properties of molecules but background scattering is a limiting factor. Su *et al.* use remote excitation by plasmons on nanowires to better access single fluorophore point spread functions for improved sensing and super-resolution imaging.

## Graphical abstract

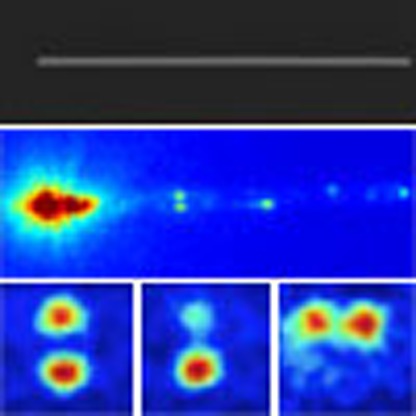

Experiments at the single-molecule level provide crucial information on the interaction between fluorophores and plasmonic nanostructures, otherwise obstructed in ensemble measurements, which can lead to improved efficiencies in sensing^1^, energy transfer^2^ and catalysis^3^. Single-molecule emission is also instrumental to super-resolution optical imaging, which has only just begun to be extended to the imaging of metallic nanoparticles^4^,^5^,^6^. Pioneering experiments by Drexhage in the 1960s showed that the radiative rate and angular distribution of emission from molecules in close proximity to the metal (near-field regime) are modified, understood classically in terms of interference between the emitter and its own reflected field^7^. The emission yield can also be enhanced dramatically by interaction with surface plasmon polariton (SPP) resonances on the metal surface (SPPs are collective oscillations of the free electron gas at the metal surface due to coupling with optical fields)^8^.

Many different approaches have been developed to study these effects at the single-molecule level. The simplest approach is to position molecules close to planar metal layers by doping them at low density in a polymer film^9^,^10^,^11^. This experimental geometry yields high reproducibility in experiments and is tractable for theoretical studies^12^. However, plasmonic nanostructures often require more complex geometries, for example, grooves, assemblies of variously shaped/sized metal nanoparticles^13^ and so on. In another instrumental approach, a sharpened metal tip, or metal nanoparticle attached to a tip, is used to approach a single molecule embedded in a polymer matrix. By controlling the distance between the metal tip/nanoparticle and the molecule, the molecular fluorescence intensity or lifetime can be tuned^14^,^15^,^16^. In the latter approach, uncertainty surrounding the exact metal nanostructure geometry, and the complexity of both the experimental apparatus and theoretical modelling, are drawbacks. Furthermore, the low density of probes in these experiments means that considerable operation time is required to collect statistically meaningful data.

Recently, we implemented single-molecule switching fluorescence microscopy to study molecular fluorescence near metallic nanostructures^4^. This technique temporally separates the fluorescence of a group of closely spaced molecules by switching them between emissive and non-emissive states. One can then image the point spread function (PSF) of a single molecule’s fluorescence even if many other molecules, temporarily non-emissive, are present within a radius defined by the optical diffraction limit (~300 nm). Our initial work highlighted a fundamental challenge however: single-molecule fluorescence PSFs are very difficult to characterize precisely due to high background emission/light scattering from the nanoparticle itself within the diffraction-limited excitation spot. Indeed, while we could fit the majority of observed PSFs to single, two-dimensional (2D) Gaussian profiles, assuming each spot corresponded to a different single molecule, ~10% of the PSFs showed bilobal and other asymmetric shapes that could not be resolved clearly^4^. The resulting super-resolution images of the nanoparticles consistently overestimated their actual dimensions.

Here we employ a modified single-molecule switching fluorescence microscopy technique that dramatically improves the imaging of fluorescence PSFs near metal nanoparticles. The technique employs dyes that stochastically switch from emissive to dark states, allowing us to determine fluorescence PSFs on the surface of a silver nanowire and to build up super-resolution images of the nanowire using (direct) stochastic optical reconstruction microscopy ((d)STORM)^17^,^18^,^19^,^20^,^21^. More importantly, instead of directly exciting the molecules on the nanowire at the point of imaging as done previously, we employ surface plasmons propagating along a silver nanowire to excite the molecular fluorescence remotely^22^,^23^. By avoiding optical excitation at the point of fluorescence detection, nanowire background emission and molecular photodegradation are both reduced significantly. It is this combination of switching fluorescence microscopy and remote excitation (RE-SFM) that allows us to clearly resolve a surprisingly complex array of fluorescence PSFs for single molecules on the nanowire surface, including PSFs from a single molecule consisting of up to four 2D Gaussian-shaped spots. With the help of simulations, each observed PSF is attributed to a single molecule adsorbed at a specific position and orientation relative to the nanowire, and we find that the degree of complexity of the PSFs is linked to the nanoparticle dimensions. The results explain definitively the inaccuracy of super-resolution imaging of metal nanoparticles by fluorescence localization that assumes single, 2D Gaussian-shaped PSFs for single molecules. Furthermore, they suggest ways to use far-field optical imaging for rapid size sorting of metallic nanoparticles, and for determining the orientation and position of molecules adsorbed on metal nanoparticles.

## Results

### Alexa 647-labelled silver nanowires

**Figure 1:**
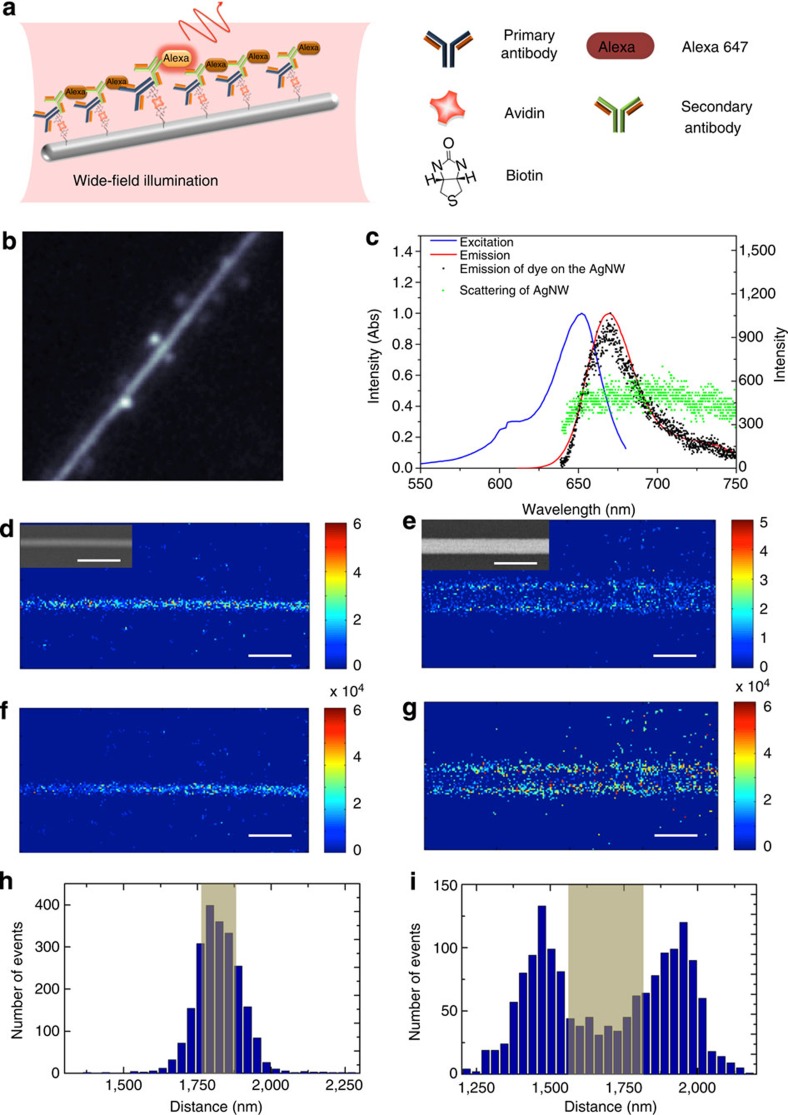
Wide-field excitation of switching localization microscopy. (**a**) Schematic illustration of wide-field excitation of the Alexa 647-labelled silver nanowire. Legends on the right denote the components for labelling the Ag nanowire (details in Methods). (**b**) The fluorescence image of an Alexa 647-labelled Ag nanowire excited by wide-field excitation in switching buffer. Strong background emission/scattering from the nanowire is clearly observable, together with bright satellite emission spots due to Alexa 647 emission. (**c**) Excitation (blue) and fluorescence (red) spectra of Alexa 647 in PBS solution, a fluorescence spectrum of an Alexa 647-adsorbed Ag nanowire in switching buffer (black dots), and an emission/Raman scattering spectrum of an Alexa-free nanowire (green dots) in switching buffer. (**d**–**g**) High-resolution reconstructed images taken with wide-field excitation (bin size 32 nm), each observed PSF was fit to a single 2D Gaussian function, with the molecule localized at the centroid of the function. Localized single-molecule density maps of a 110-nm diameter nanowire (**d**) and a 250-nm diameter nanowire (**e**) and localized single-molecule intensity histograms of the 110-nm nanowire (**f**) and 250-nm diameter nanowire (**g**). The insets in **d** and **e** display the SEM images of the corresponding nanowires. (**h**) and (**i**) are histograms of the localized single-molecule density maps along cross-sections perpendicular to the long axis of the nanowires displayed in **d** and **e**, respectively. The grey shaded areas represent the actual nanowire cross-sections measured by SEM. Scale bars, 1 μm.

Metallic nanowires are an important class of plasmonic nanostructures, serving as resonators^24^, sub-diffraction limit plasmon waveguides^22^,^25^,^26^,^27^ and templates for highly integrated structures such as nanoantennas^28^ and photonic crystals^29^. The silver nanowires used here have diameters from 100 to 300 nm, possess five-fold symmetry (penta-twinned crystals) with a pentagonal cross-section^30^,^31^ and were synthesized using a polyol synthesis protocol^30^. Fluorescent dyes were attached to the nanowire surface via a bulky bioconjugation to prevent direct (physical) contact between fluorophore and metal. More specifically, secondary antibodies labelled with fluorescent dye were linked to the metal surface via thiol-biotin/avidin/biotinated primary antibody (see Fig. 1a and further details in the Methods section). In our experiments, the dye Alexa 647 was chosen as the label, since excitation light intensity can modify its rate of stochastic switching between fluorescent and non-fluorescent states in appropriate buffer conditions^21^ (specifically the yield of triplet formation and other long-lived non-fluorescent intermediates is modified). The distance between the dyes and the surface of the nanowires is estimated to be 7–15 nm (refs 32, 33), which is in the range where both enhancement and quenching of molecular fluorescence can occur by interaction with the metal^7^,^16^,^34^,^35^. Here fluorescence lifetime studies show that the decay rates of Alexa 647 are indeed enhanced (shortened lifetime) in close proximity to the metal (Supplementary Figs 1 and 2b), with an average lifetime of 0.51 ns bound to the nanowire compared with 1.01 ns in buffer. Three mechanisms can enhance decay for excited molecules at these molecule-metal separations, energy transfer to non-optical surface modes of the metal, an enhanced radiative rate due to in-phase reflection of the molecules emission by the metal and finally coupling to SPPs on the nanowire^7^. In the latter case, the SPPs can rescatter into the far-field at the ends of the nanowire if they are not dissipated by Ohmic losses. The relative quantum yield of each of these processes depends greatly on the specific distance/orientation of the bound molecule; however, the ability to observe bright single Alexa 647 emission on the nanowires here suggests that neither energy transfer to non-optical modes, or coupling to SPPs, is a dominant relaxation mechanism. Indeed it is the direct radiative relaxation of the molecules into the far-field, with emission direction perturbed by proximity to the nanowire, which is the focus of this work.

### Visualizing PSFs by wide-field excitation

In Fig. 1b, a fluorescence image of an Alexa 647-labelled nanowire during wide-field excitation (633 nm) is shown. Wide-field illumination was employed for exciting the nanowire and surrounding areas and the laser power was carefully adjusted (~2 kW cm^−2^) to ensure that only a subset of fluorophores was activated at any time, despite the nanowire being densely and homogeneously labelled (see Supplementary Fig. 2a). The fluorescence spots appear most clearly as satellites along the nanowire structure, which also exhibits significant emission (Fig. 1b). A time-averaged emission spectrum from an Alexa 647-bound silver nanowire clearly matches that of Alexa 647 in buffer solution (Fig. 1c). Conversely, the time-averaged spectrum from an Alexa-free nanowire is broad and structureless, typical of metallic nanostructures and attributed to Raman scattering and/or luminescence of the metal or other impurities (Fig. 1c). This broad emission cannot be filtered from, and is clearly of a similar order of magnitude to, the Alexa emission during wide-field excitation, a fundamental problem for the precise measurement of fluorescence PSFs.

Indeed under these conditions, one can attempt to visualize the pure Alexa 647 fluorescence events by a background subtraction method. Since the molecules are stochastically switching between bright and dark states, the molecular emission spot will not appear in the following (perhaps second or third) frame, allowing reasonably accurate background subtraction (see ref. 4 for full details). This process is most effective for relatively intense fluorescence events; however, the extracted PSFs appear as single spots of varying broadness and symmetry, any further complexity having been washed out by the high background emission from the nanowire. Nevertheless, high-resolution reconstruction imaging of nanowires under wide-field excitation was undertaken, fitting the observed emission PSFs as best as possible to single, 2D Gaussian-shaped functions and localizing the molecule at the centroid of the function (Fig. 1d–g). For a 110-nm diameter nanowire both the density map (Fig. 1d) and the intensity histograms (Fig. 1f) of localized single-molecule emission have the expected nanowire shape, but the cross-section of both images predict a diameter of ~205 nm (Fig. 1h). For a 250-nm diameter nanowire, both the density map (Fig. 1e) and the intensity histogram (Fig. 1g) completely fail to reproduce the form of the nanowire; instead, two separate lines of molecular localization build up, separated by ~500 nm (Fig. 1i).

### Visualizing PSFs by remote excitation

**Figure 2:**
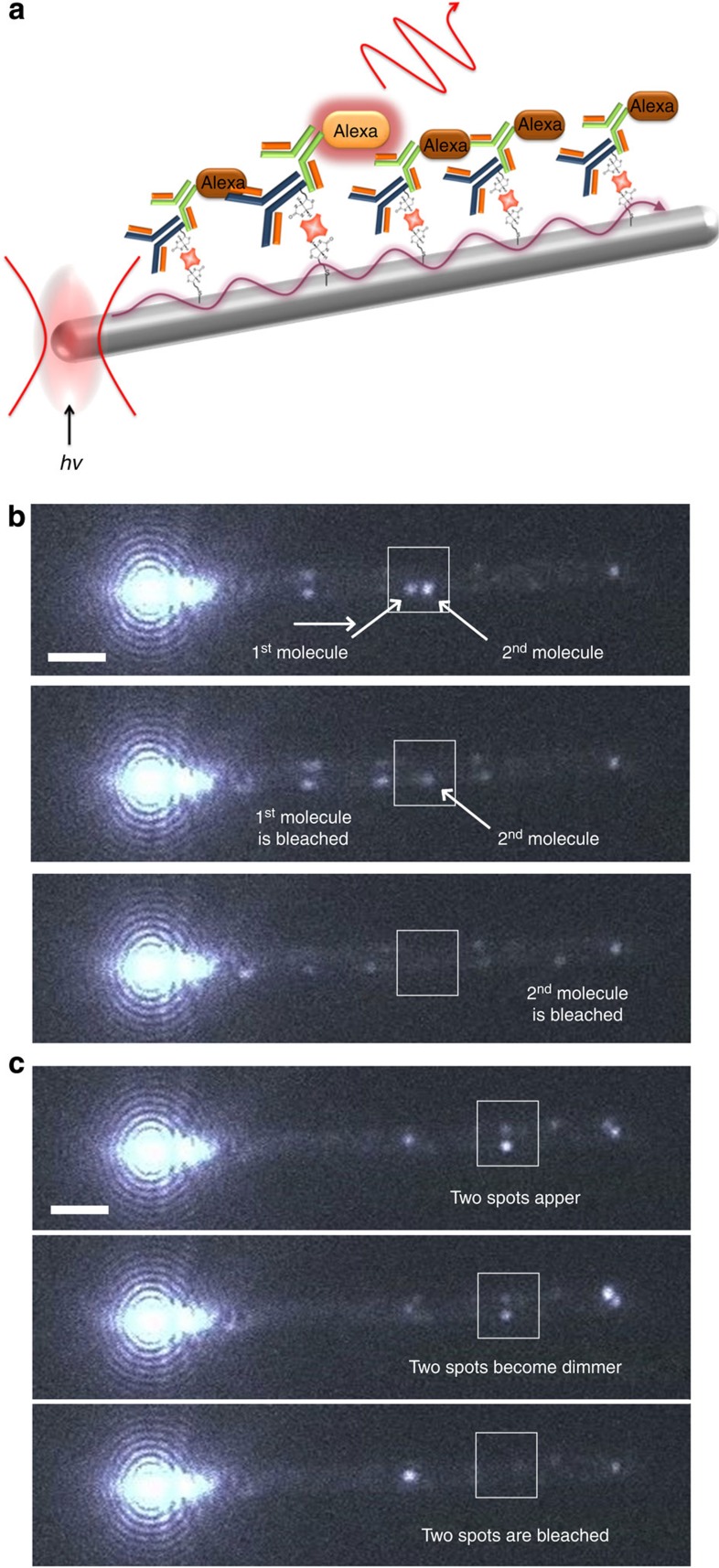
Remote excitation of switching localization microscopy. (**a**) Schematic illustration of RE-SFM, the Ag nanowire is irradiated at the left end with focused laser, launching SPPs that propagate along the surface of the nanowire (purple curly arrow), and excite the surface-bound Alexa molecules. (**b**) RE-SFM images showing two fluorescence spots along the nanowire (enclosed by the white square) bleaching separately. (**c**) RE-SFM images showing two fluorescent spots along the nanowire (enclosed white square) appearing, dimming and disappearing (blinking off) at the same time in subsequent images. Scale bars, 2 μm.

The same experiment was then conducted employing our remote excitation technique, where the 633-nm excitation laser is focused at the end of the nanowire to launch SPPs which propagate >10 μm along the wire to excite Alexa molecules adsorbed within the SPP near-field (Fig. 2a)^22^. Again, the excitation intensity was modulated so that only a few molecules were emitting at any time (~500 kW cm^−2^). The resulting fluorescence images (Fig. 2b,c) show bright emission due to scattered excitation light at the end of the nanowire that is excited; however, further along the nanowire, the contrast with the wide-field image (Fig. 1b and Supplementary Movie 1) is remarkable. Now the Alexa 647 emission stands out clearly against the much weaker background emission from the nanowire. The average fluorescence intensity observed from single molecules of Alexa 647 during RE-SFM imaging decayed exponentially with distance from the laser focus (decay length ~8 μm) as expected for excitation by propagating SPPs (Supplementary Fig. 3b). Furthermore, the excitation of fluorescence by Rayleigh-scattered laser light is ruled out for fluorescent spots >2 μm from the excited end of the wire (Supplementary Fig. 4).

**Figure 3:**
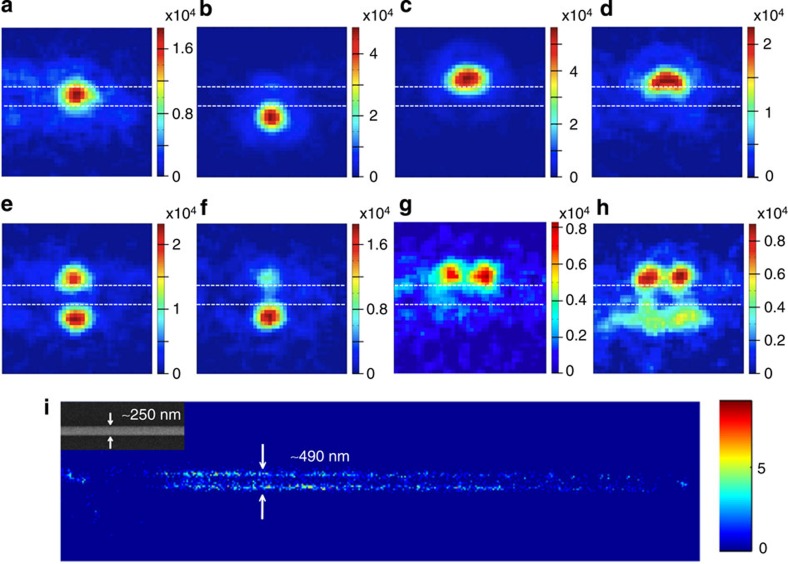
Multi-spot fluorescence PSFs of single molecules near a Ag nanowire. A series of PSFs of single-molecule fluorescence in the vicinity of a silver nanowire (~250 nm diameter) observed during RE-SFM measurement (image sizes: 1.94 × 1.94 μm^2^, the parallel dashed lines indicate the edges of the nanowire). (**a**) A 2D Gaussian-shaped PSF at the middle of the nanowire. (**b**) A 2D Gaussian-shaped PSF at the side of the nanowire. (**c**,**d**) Elongated PSFs at the side of the nanowire. (**e**) A symmetric PSF with two 2D Gaussian-shaped spots on opposite sides of the nanowire. (**f**) An asymmetric PSF with two 2D Gaussian-shaped spots on opposite sides of the nanowire. (**g**) A symmetric PSF with two 2D Gaussian-shaped spots along one side of nanowire. (**h**) A PSF with four 2D Gaussian-shaped spots, two on either side of the nanowire. (**i**) High-resolution (bin size 32 nm) reconstructed single-molecule localization density map of an ~250-nm diameter nanowire from RE-SFM (SEM image inset) for which a molecule is assumed to be localized at the centroid of each fluorescence spot observed. PSFs were fit by single- or multi-component 2D Gaussian functions and a ‘molecule’ localization recorded at the centroid of each peak (that is, a two-spot PSF leads to the localization of two ‘molecules’). The ‘bare’ spot in the image at the left end of the wire is where the excitation laser was focused, background emission and scattering overwhelm Alexa 647 emission in this region. The spots on the far left of the image, and far from the nanowire position, are due to residual Alexa 647 that was not washed completely off the substrate.

Careful study of successive RE-SFM images (integration time 50 ms) lead us to an important realization: while many emission spots blink on and off individually as expected if they are due to separate single molecules (Fig. 2b), other closely spaced spots blink on and off synchronously suggesting they are in fact complex, multi-spot PSFs due to a single dye (Fig. 2c). In Fig. 3, a range of complex PSFs attributed to single molecules observed over time on a section of a silver nanowire (diameter ~250 nm) under remote excitation are shown. Fluorescence spots are generally observed at the edge of the nanowire, in line with the wide-field measurements, but now their complexity is clear: symmetric single-spot PSFs (Fig. 3a,b); elongated single-spot PSFs (Fig. 3c,d); symmetric two-spot PSFs appearing at opposite sides of the wire (Fig. 3e); asymmetric two-spot PSFs appearing at opposite sides of the nanowire (Fig. 3f); two-spot PSFs on one side of the nanowire (Fig. 3g); and four-spot PSFs with two spots on either side of the nanowire (Fig. 3h). In Fig. 3i, we demonstrate that fitting these complex PSFs with the normal assumptions of reconstruction imaging, that a separate molecule is localized at the centroid of each fluorescent spot observed, leads to an inaccurate reconstruction image of an ~250-nm nanowire. Each complex PSF was fitted to a single- or multi-component 2D Gaussian function and a molecular localization recorded at the centroid of each Gaussian peak (that is, four ‘molecules’ were localized for a single four-spot PSF). The reconstructed image fails to reproduce the nanowires true form in a similar way as for the high-background wide-field excitation studies in Fig. 1g.

### Simulating fluorescence PSFs

**Figure 4:**
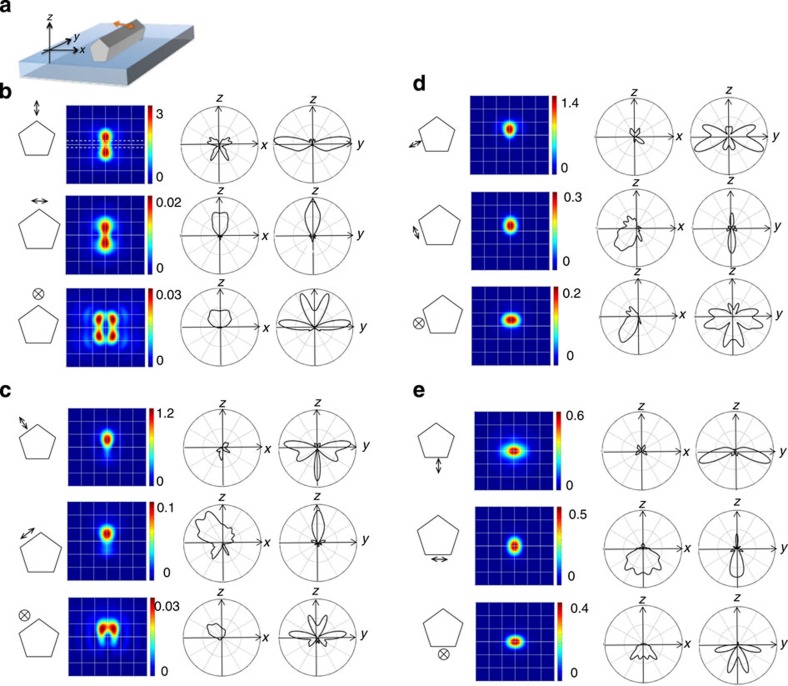
Simulated fluorescence PSFs for single molecules on Ag nanowires. (**a**) Sketch of the simulation geometry, the double-headed arrow indicates the position and orientation of the oscillating electric point dipole that simulates a single fluorophore in the vicinity of the nanowire (~280 nm diameter). (**b**–**e**) Far left: the position and orientation of the point dipole (indicated by the double-headed arrow) relative to the pentagonal cross section of the silver nanowire. Left centre: the simulated far-field emission PSF colour map for the point dipole imaged in the sample plane (nanowire edges indicated by dashed lines in (**b**), top colour map). The image size is 3 × 3 μm^2^. Right centre: polar plot of the angular dispersion of point dipole emission in the *xz* plane, Far right: polar plot of the angular dispersion of the point dipole emission in the *yz* plane.

The distortion of the angular distribution of a molecules emission by its proximity to a metallic tip or nanorod has been treated theoretically and observed experimentally^36^,^37^, and we previously simulated the angular distortion of single-molecule emission in the vicinity of a silver nanowire^4^. In Fig. 4, we extend this modelling to show the result of the angular distortion when the sample plane is imaged in the far-field by our optical microscope, that is, the simulated fluorescence PSF. The finite-difference time-domain method was used to numerically simulate the far-field emission. We considered a pentagonal nanowire immersed in the switching buffer solution (refractive index ~1.33) on a dielectric (glass) substrate (Fig. 4a), and of similar diameter to the nanowire imaged experimentally in Fig. 3. A single fluorescing molecule was modelled as an oscillating electric point dipole located 10 nm from the metal surface. As depicted on the left side of Fig. 4b–e by a double-headed arrow, this dipole is set to be oriented normal, or parallel to, each facet and the apex of the nanowire. Far-field photon flux into the glass side was monitored and the corresponding PSF at the image plane was calculated by Fourier transformation of the far-field projection by taking into account the numerical aperture (NA) of the objective lens used in the experiment (NA=1.3).

The simulated far-field PSFs (colour maps, Fig. 4b–e) reflect the modified angular distribution of single-molecule emission (simulated in the left polar plots for the sample *xz* plane and in the right polar plots for the *yz* plane in Fig. 4b–e) and show good agreement with the various experimentally observed PSFs in Fig. 3. The simulations suggest that single-spot, near Gaussian-shaped fluorescence PSFs originate from locating the dipole at facets in the lower half space of the nanowire (near the glass substrate) with their centroid position depending at which facet the dipole locates (see Fig. 4d,e). These correspond well with the experimentally observed PSFs in Fig. 3a,b. When the dipole is oriented parallel to the long axis of the nanowire, the simulated Gaussian-like PSF is elongated (Fig. 4d,e), as observed experimentally in Fig. 3c,d.

Simulated PSFs with multiple spots result from locating the dipole at a facet in the top half space of the nanowire (the buffer solution side). PSFs with two spots perpendicular to the nanowire long axis, frequently observed in RE-SFM images (Fig. 3e,f), are also found in the numerical simulation (Fig. 4b,c). The symmetry of this type of simulated PSF is sensitive to the dipole location. When the dipole is located near the top of the nanowire, the pattern is symmetric, while when the dipole is located at the side facet, an asymmetric PSF is predicted. The simulated PSF splits into four spots when the dipole is placed on top of the nanowire, with its moment parallel to the long axis of nanowire (see bottom of Fig. 4b and compare with the experimental result in Fig. 3h). The intensity of the four spots is sensitive to the position and the angle of the dipole relative to the nanowire long axis (see Supplementary Fig. 5). When the dipole is placed at a side facet in the top half space with its moment parallel to the long axis as depicted at the bottom in Fig. 4c, the predicted PSF consists of two spots appearing on one edge of the nanowire, a PSF that is also observed experimentally (Fig. 3g).

It is worth noting that the simulations predict single-molecule fluorescence collection efficiency to also be strongly modified depending on the location and orientation of the molecule with respect to the nanowire. For example, intense PSFs should be observed for the molecule position and orientation shown in the middle row of Fig. 4e, where emission is scattered towards the detection path (directly along the *z* axis in the glass direction, see polar plots). On the contrary, for molecules in the same position but with different orientation, such as simulated in the top row of Fig. 4e, emission is primarily scattered away from the detection path resulting in weaker PSF intensity.

### Simulating super-resolution reconstructed images

**Figure 5:**
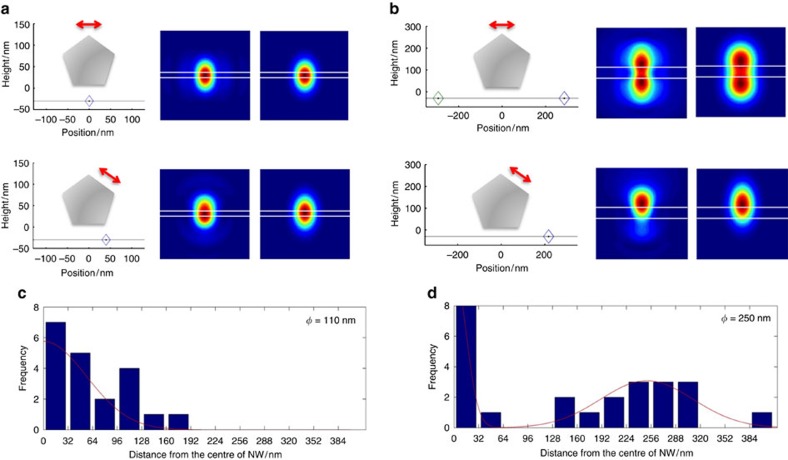
Localization of simulated fluorescence PSFs. (**a**,**b**) Simulated PSFs of a point dipole oriented perpendicular to the long axis of the nanowire and placed at the apex or on the adjacent facet of a 110-nm diameter (**a**) and 250 nm diameter (**b**) nanowire (colour maps, centre, the white lines indicate the edges of the nanowire). The PSFs are fit by single or multi-component 2D Gaussian functions (colour maps, right) and ‘molecule’ localizations recorded at the centroid of each Gaussian peak (that is, assuming that each fluorescent spot observed is from a separate molecule at its centroid). For instance, fitting the two-spot PSF at the top of **b** yields two localized ‘molecules’ (localization positions indicated by the diamonds in the schematic cross-section of the nanowires on the left) (**c**,**d**) Cross-sectional histograms of ‘molecule’ localizations perpendicular to the nanowire long axis for the simulated PSFs of the full range of molecular adsorption positions and orientations shown in Fig. 4 for nanowires of 110 nm (**c**) and 250 nm (**d**), diameter respectively. The histograms are fit by Gaussian distributions (red line) indicating a full width at half maximum, and thus predicted nanowire diameter, of ~160 nm for the hypothetical 110 nm diameter nanowire (**c**) and two lines separated by ~500 nm surrounding a line centred along the nanowire long axis for the hypothetical 250 nm diameter nanowire (**d**). Scale bars, 500 nm.

As a further confirmation of the robustness of these simulations, the full set of simulated PSFs for the molecule positions and orientations shown in Fig. 4 was determined for nanowires with theoretical diameters of 110 and 250 nm. Each of these simulated PSFs was then fit to a single- or a multi-component 2D Gaussian function and a simulated ‘molecule’ localization recorded at the centroid of each Gaussian peak (Fig. 5a,b). In this way, simulated high-resolution reconstructed images were built up of the two nanowires with hypothetical homogenous coverage of dye, and assuming that a ‘molecule’ is located at the centroid of each simulated fluorescent spot. While the simulated PSFs used represent only a subset of the full range of possible positions/orientations of molecules around the nanowire, the histograms of the cross-section of these simulated localization maps reproduce very well those determined experimentally in Figs 1 and 3, as well as their inaccuracies (Fig. 5c,d). For the simulated 110-nm diameter nanowire, a width of 224 nm is obtained from the histogram. For the simulated 250-nm diameter nanowire, two lines of localization separated by ~500 nm are obtained just like in experiments. However, a line of molecular localization exactly along the nanowire is also predicted, which is not observed as clearly experimentally in either wide-field or RE-SFM imaging, likely due to photodegradation of the dyes in direct contact with the glass substrate.

## Discussion

This work explains the origin of inaccuracies associated with super-resolution reconstruction imaging of metal nanowires, which are clearly present here even for nanowire diameters well below the diffraction limit. Even overcoming the challenges of high background emission washing out the true form of the PSF by using remote excitation, the normal preconception in super-resolution imaging, that each fluorescence spot is due to a single molecule at its centroid, leads to serious errors. Indeed there is a circularity in the task at hand. Since molecular PSFs are so sensitive to the nanowire shape, accurate reconstruction imaging by correctly fitting each PSF to extract the true molecular position may only be possible with a priori knowledge of the nanowire dimensions. Recent development of integrated scanning electron microscopy (SEM)/fluorescence microscopy alleviates this task^38^. The complex and often multi-spot nature of the PSFs must also be carefully considered in other single-molecule fluorescence studies of, for instance, catalysis on metal nanowires^39^,^40^. Counting a single turnover event associated with a four-spot PSF as four separate events would skew statistics significantly and also give misleading conclusions about the position of active sites on the wire.

Nevertheless, the good correspondence between the experimental PSFs and simulated PSFs suggests ways in which one might make rapid predictions of, for instance, nanoparticle dimensions or the position and orientation of single molecules on a nanoparticle, via far-field optical imaging. Of most utility here are the multi-spot PSFs whose shapes are strongly and characteristically effected by, for example, nanowire diameter. The complex PSF for a molecule at the apex of the nanowire (buffer solution side) with transition dipole perpendicular to the long axis simulated in Fig. 5a,b is a good example of this sensitivity. One can define a threshold wire thickness above which PSFs with two clear spots can be observed instead of one, the appearance of which would constitute an optical assay of a group nanowires for those with diameter, for example, >150 nm. We stress that this assay, and those mentioned below, require a priori knowledge of the nanoparticle(s) at hand to be successful, that is, one must know that one is dealing with a sample of nanowires of various thicknesses.

The symmetry of the four-spot PSF of a molecule at the apex of the nanowire (solution side) with transition dipole parallel to the nanowire axis, and it’s sensitivity to adsorption position and angle, has already been discussed (Supplementary Fig. 5). One might also assay any preferential orientation of single molecules on the apex of the nanowire, by looking for departures from the expected 2:1 appearance ratio of the relevant two- and four-spot PSFs (Fig. 4b), though care would need to be taken here to consider also the relative intensities of these PSFs and their probability of detection above the background.

Finally, the very appearance of distinct, complex PSFs already gives crucial information regarding the timescale of molecular rotations versus the timescale of the experiment. If the rotation of the molecule is much faster than the image integration time (typically 50 ms), the images would be a blurred mixture of many different PSFs. It is possible that an apparently single PSF is observed for a freely rotating dye, since the fluorescence intensity collected by the objective lens depends strongly on the molecular orientation (see Fig. 4), and a very bright PSF might then dominate the image. However, the experimentally observed four- and two-spot PSFs are predicted to have relatively weak intensity (Fig. 4b, bottom, and Fig. 4c, bottom, respectively) and thus would be obscured if the dyes were rotating freely here. The hindered mobility for the dyes bound to the nanowire here is likely due to the strong binding affinity of the biolinkers and their dense packing on the nanowire surface (Supplementary Fig. 2a).

In conclusion, the employment of remote excitation switching fluorescence microscopy on Alexa 647-labelled silver nanowires allowed us to resolve precisely the fluorescence PSFs of single molecules above the background emission of the nanowire. Most importantly, multiple fluorescence spots were seen to blink synchronously, indicating they constitute the PSF of a single molecules emission. Numerical simulations confirm that these multi-spot PSFs are particularly sensitive to the fluorophores position and orientation of adsorption on the nanowire, and dependent further on the dimensions of the nanowire itself. While this is problematic for accurate super-resolution reconstruction imaging of metallic nanowires, it highlights the need for rigorous checks to ensure correct PSF fitting for molecules near metallic nanoparticles generally. With such knowledge, the complex PSFs could be used predictively in far-field imaging assays of nanoparticle size/shape, or of molecular adsorption site/orientation, given some prior knowledge of the nanoparticle sample. This work is another step in unravelling and understanding the complex interactions between molecules and plasmonic nanostructures with implications for improving the efficiency of photovoltaics, photocatalysts and (bio)chemical sensors.

## Methods

### Preparation of metallic nanowires

Silver nanowires were prepared using a modified polyol synthesis^30^. In brief, a 3-ml ethylene glycol (anhydrous, Sigma-Aldrich) solution of silver nitrate (100 mM) (99.9999% Sigma-Aldrich) and a 3-ml ethylene glycol solution of poly(vinylpyrrolidone) (Mw~40,000, Sigma-Aldrich; 600 mM) were injected at a rate of 50–200 μl min^−1^ into 5 ml of ethylene glycol and refluxed at 160 °C for more than 1 h. During the injection, the solution was irradiated with white light (400–800 nm) to induce a high yield of nanowire formation^30^. Nanowires were separated from small nanoparticles and short nanowires by filtering the suspension using a poly(propylene) filter with 2.5-μm pores (Millipore). The nanowires were finally redispersed in ethanol.

### Nanowire surface functionalization

The silver nanowires were first resuspended in 0.5 ml ethanol solution of biotin-terminated poly(ethylene glycol) (~2.5 mM, Nanoscience Instruments). Then the mixture was bubbled with argon gas for two minutes and kept shaking overnight. The suspension was centrifuged at 4,000 r.p.m. and the supernatant exchanged with pure ethanol. This process was repeated twice. The biotinated nanowires were then kept in 0.5 ml of PBS solution of avidin (~0.6 mg ml^−1^) at 4 °C for 2 h, and purified twice using centrifugation at 4,000 r.p.m. Those samples were immersed in PBS buffer of biotinated HIS-tag antibody (IgG1, SM1693B, Acris Antibodies; ~3 μg ml^−1^) at 4 °C for 2 h and the excess antibody was removed. Finally, the nanowires with antibody were further functionalized with Alexa 647 Goat Anti-Mouse IgG (Life Technologies) in PBS buffer at 4 °C for 2 h and the excess antibody removed. The silver nanowires were then drop-cast onto a cover slip which had been silanized by (3-aminopropyl)trimethoxysilane (Sigma-Aldrich). After that, the sample was kept in a sealed chamber filled with switching buffer, PBS (pH 7.4), containing oxygen scavenger (0.5 mg ml^−1^ glucose oxidase (Sigma-Aldrich), 40 mg ml^−1^ catalase (Roche Applied Science), 10% w/v glucose) and 50 mM β-mercaptoethylamine^20^.

### Switching fluorescence microscopy

The wide-field fluorescence microscope consisted of an inverted optical microscope (Ti-U, Nikon) equipped with a × 100 oil immersion objective (NA=1.3, CFI Plan, Nikon) and a cooled electron multiplying charge-coupled device (CCD) camera (ImagEM, Hamamatsu). The 632.8-nm light from a He-Ne laser (1145P, JDSU) was used as the excitation source. For wide-field irradiation, collimated laser light was focused at the back focal plane of the objective. For remote excitation studies, collimated laser light was guided to the objective to achieve a diffraction-limited focus at the sample plane. Emission was collected by the same objective and imaged by the CCD after passing through a dichroic mirror (z633rdc, Chroma Technology Co.) and additional spectral filters (HQ655LP, Chroma Technology Co). λ/4 and λ/2 waveplates were used to generate a circularly polarized excitation beam. The image was further magnified 3.3 times with a camera lens before the CCD camera, resulting in a maximum field of view of 24.6 × 24.6 μm^2^ (48 × 48 nm^2^ per pixel).

### Numerical calculation of far-field scattering

The angular distribution of the far-field scattering of molecular fluorescence in the vicinity of the metallic nanowire was numerically calculated using the finite-difference time-domain method (Lumerical Solutions Inc., Vancouver, Canada). The silver nanowire was modelled as a pentagonal prism with various diameters. The tabled values of Johnson and Christy were used for the dielectric function of silver. The nanowire was placed on a dielectric slab with refractive index 1.5 (simulating the glass substrate) and surrounded by an infinite dielectric environment with refractive index 1.33 (simulating the buffer). A single fluorescent molecule was modelled as an oscillating electric point dipole placed at 10 nm from the nanowire surface.

### SEM measurements

After fluorescence measurements on a specific nanowire, the sample was washed with MilliQ water several times and coated with a thin layer of gold using an auto-fine coater (JEOL JFC-1300). SEM images were then taken of the same nanowire using a FEI Quanta FEG 250.

## Author contributions

L.S. and H.U. conceived and designed the experiment. L.S. and G.L. carried out optical measurments. L.S., B.K., H.Y. and M.B.J.R. performed correlative measurements. L.S., E.F., C.C. and P.V.D. performed the time-resolved measurements. S.R. and H.U. wrote the analysis software. L.S. and H.U. analysed the data and simulation. L.S., H.U. and J.A.H. wrote the manuscript with contributions from all authors.

## Additional information

**How to cite this article:** Su, L. *et al.* Visualization of molecular fluorescence point spread functions *via* remote excitation switching fluorescence microscopy. *Nat. Commun.* 6:6287 doi: 10.1038/ncomms7287 (2015).

## Supplementary Material

Supplementary Figures and Supplementary References Supplementary Figures 1-5 and Supplementary References.

Supplementary Movie 1 Remote excitation single-molecule fluorescence imaging of silver nanowire labelled with Alexa 647.
