# Supplementary material for: Visualization of molecular fluorescence point spread functions via remote excitation switching fluorescence microscopy
Source: Nat Commun. 2015 Feb 17;6:6287. doi: 10.1038/ncomms7287 (PMC4339893; doi:10.1038/ncomms7287)
Supplement: Supplementary Figures and Supplementary References — Supplementary Figures 1-5 and Supplementary References. [file ncomms7287-s1.pdf]

Supplementary Figures

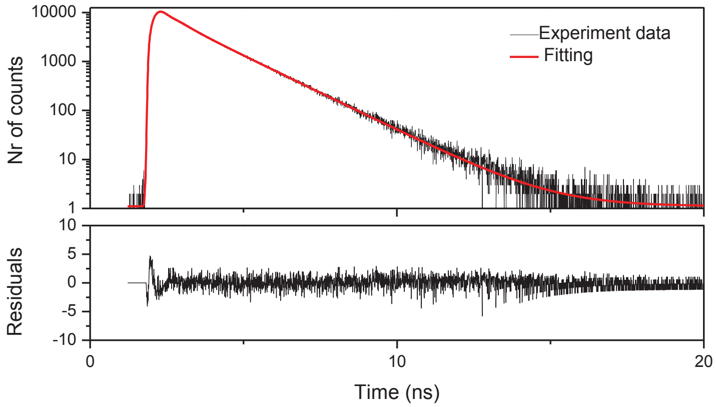

Supplementary Figure 1| Fluorescence decay of secondary antibody-bound Alexa 647 dye. Decay was recorded in PBS buffer, and best fit by a bi-exponential function with the fast and slow time constants of 0.4 ns (39%) and 1.4 ns (61%) respectively. The residuals are plotted.

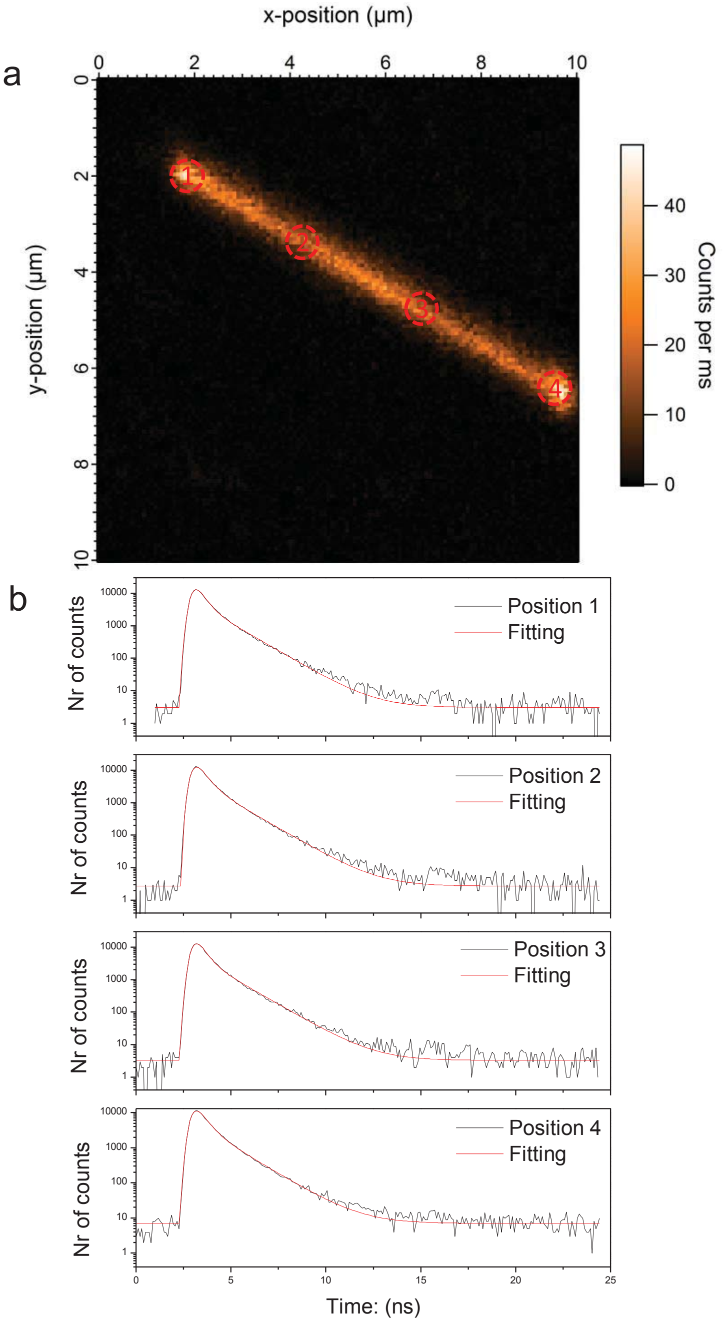

Supplementary Figure 2| Fluorescence properties of Ag nanowire-bound Alexa 647 dye. (a) Confocal scanning fluorescence image of an Ag nanowire in switching buffer. Excitation power was adjusted so that many Alexa molecules were emitting at any time within the focused laser spot and the long-time averaged fluorescence image confirms that labelling of the dye is rather homogeneous across the whole wire (b) Lifetime measurements in different positions along the nanowire were carried out (positions 1, 2, 3, and 4 as indicated in Supplementary Fig. 2a). In all these positions, bi-exponential decay kinetics were observed and fitting the data yielded average lifetimes of 0.34$\pm$ 0.05 ns (82$\pm$3%) and 1.28$\pm$0.03 ns (18$\pm$3%). The shortening of the fluorescence lifetime of the Alexa 647 in the vicinity of the metal nanoparticle observed here (average lifetime of 0.51 ns bound to the nanowire compared to 1.01 ns in buffer) is in agreement with the work of others[1, 2]. The excited molecules non-radiative decay rate can be enhanced *via* energy transfer to non-optical surface modes of the metal and by coupling to SPPs on the wire, while its radiative decay rate can be enhanced by in-phase reflection of the emission by the metal surface.

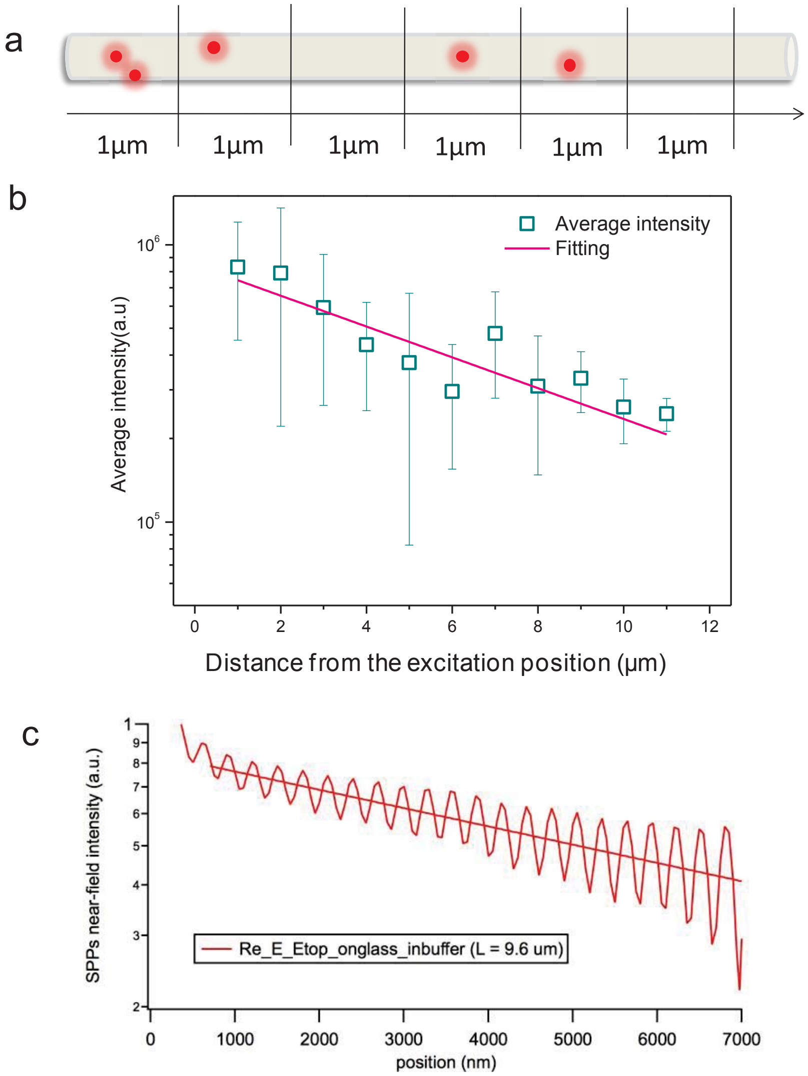

Supplementary Figure 3| Distance dependence of remote excitation fluorescence. (a) Schematic of a silver nanowire, dissected into 1 μm segments from the end where focused laser excitation occurs (left end), the red dots represent the fluorescence from single Alexa 647 molecules. (b) The average fluorescence intensity of single Alexa 647 molecules from each segment following remote excitation. The detected intensity decays exponentially with distance from the point of excitation. The red line is an exponential fit, yielding a 1/*e* decay length of ~8 μm. (c) FDTD calculation (Lumerical Inc) of SPP decay along a silver nanowire. The silver nanowire was modeled as a pentagonal prism with flat ends in an infinite dielectric environment with refractive index of 1.333 (representing the buffer). The diameter and length of the wire was set to be 300 nm and 7 μm respectively. The tabulated values of Johnson and Christy were used for the dielectric function of silver (predicted SPP 1/*e* decay length ~9 μm).

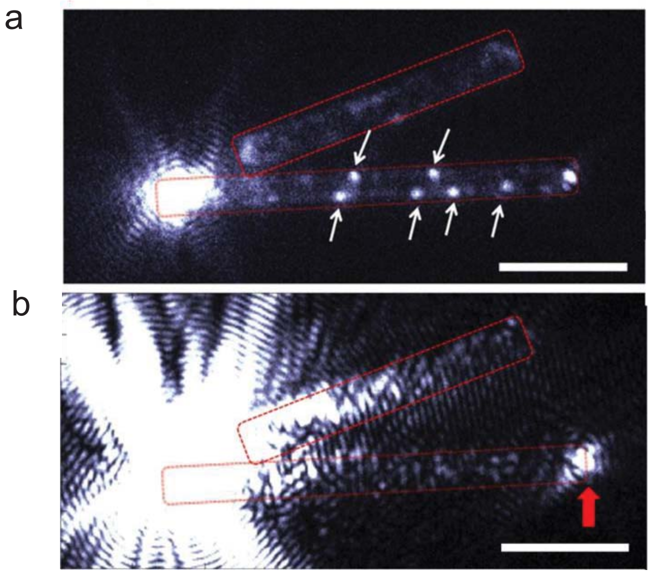

Supplementary Figure 4| Fluorescence and Rayleigh scattering images of remote excitation. (a) A typical image of remote excitation switching fluorescence microscopy (RE-SFM), performed on one of two adjacent nanowires. Laser excitation was focused on the left end of the nanowire at the bottom of the image and white arrows indicate the Alexa 647 fluorescence spots. Red dotted lines show the profile of the two nanowires, and the scale bar is 4 μm. It is clear that bright spots of Alexa 647 fluorescence are observed only on the irradiated nanowire (b) A Rayleigh scattering image of the two nanowires during the same experiment. The red arrow points to the distal end of the excited nanowire where SPPs out-couple to far-field radiation. The red dotted lines show the profile of the two nanowires, and the scale bar is 4 μm. These images prove that propagating SPP modes on the nanowire are indeed efficiently launched and scatter to far-field radiation at the opposite end of the nanowire. They also prove that it is these propagating SPPs that are the primary source of excitation of the surface-bound Alexa dyes adsorbed >2 μm away from the end of the wire being excited.

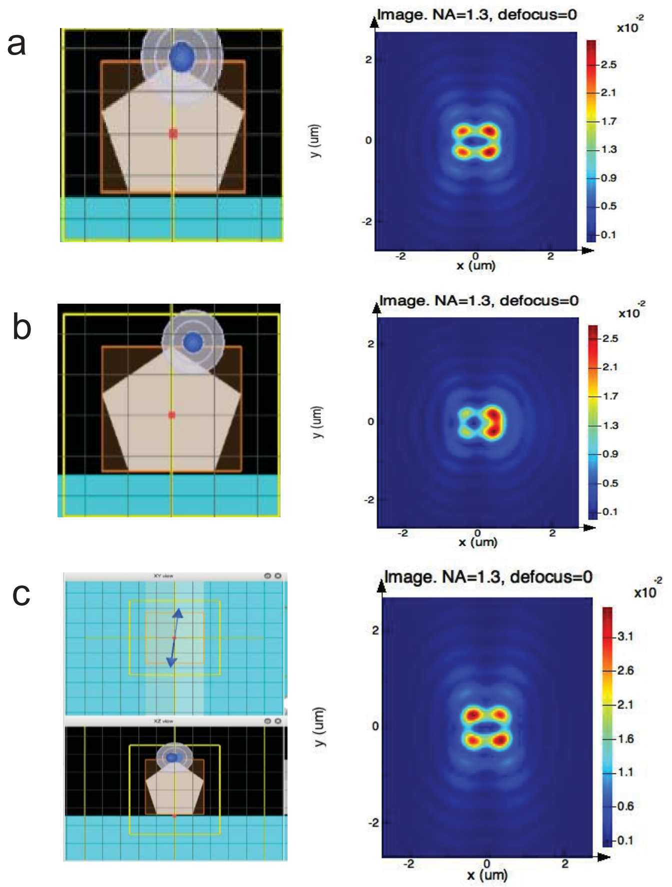

Supplementary Figure 5| Simulated PSFs of a radiating point dipole on top of a silver nanowire. Simulated fluorescence point spread functions (right) corresponding to the position and orientation of a radiating point dipole (indicated by the blue, double-headed arrow) relative to a Ag nanowire. A four spot fluorescence PSF is predicted by FDTD simulations when the dye is located on top of the nanowire with its transition dipole moment parallel to the long axis of nanowire. The symmetry of this PSF is however very sensitive to the dye location. If the dye is moved to the right of the position directly above the nanowire, the two spots on the left side of the PSF diminish, and the two spots on the right side of the PSF brighten. As the molecule is moved further to the right, the PSF experiences further distortion, and the two spots on each side start to merge. The symmetry of the intensities of the four spots is also sensitive to the angle of the dipole relative to the nanowire long axis. The simulation predicts that if the dye is located directly above the nanowire, but with its transition dipole angled slightly away from the long axis of nanowire, the PSF will undergo diagonal distortion. The intensities of spots on the top-left and bottom-right increase, while the intensities of spots on top-right and bottom-left decrease.

Supplementary References

1. Anger, P., Bharadwaj, P. & Novotny, L. Enhancement and quenching of single-molecule fluorescence. *Phys. Rev. Lett.* **96**, 113002 (2006).
2. Kuhn, S., Hakanson, U., Rogobete, L. & Sandoghdar, V. Enhancement of single-molecule fluorescence using a gold nanoparticle as an optical nanoantenna. *Phys. Rev. Lett.* **97**, 017402 (2006).
